# Enhancing wheat resilience to salt stress through an integrative nanotechnology approach with chitosan proline and chitosan glycine

**DOI:** 10.1038/s41598-025-91496-w

**Published:** 2025-04-01

**Authors:** Fatemeh Gholizadeh, Gholamreza Gohari, Magda Pál, Gabriella Szalai, Imran Khan, Tibor Janda

**Affiliations:** 1 Department of Plant Physiology and Metabolomics, Agricultural Institute, HUN-REN Centre for Agricultural Research, Martonvásár, 2462 Hungary; 2 Department of Horticultural Sciences, Faculty of Agriculture, University of Maragheh, Maragheh, 55181-83111 Iran

**Keywords:** Salt stress, Wheat, Nanocomposites, Antioxidant enzymes, Gene expression, Biotechnology, Nanobiotechnology, Nanoparticles

## Abstract

Salt stress significantly limits wheat production worldwide, jeopardizing food security and sustainable agriculture. Developing strategies to enhance wheat’s resilience to salinity is critical for maintaining yield in affected regions. This study investigates the potential of chitosan-proline (Cs-Pro) and chitosan-glycine (Cs-Gly) nanoparticles in mitigating salt stress in salt-tolerant Heydari and salt-sensitive Sepahan wheat cultivars, with a special question on genotype-dependent differences. Plants were treated with nanoparticles at concentrations of 0, 200, and 400 mg L⁻¹ under salt stress levels of 0, 200, and 400 mM NaCl. The salt-tolerant Heydari cultivar exhibited superior adaptability to saline conditions, in addition reacted more positively to nanoparticle treatments. Results demonstrated significant physiological improvements, including increased relative water content (RWC), enhanced chlorophyll content and elevated proline levels, especially after 400 mg L⁻¹ Cs-Pro treatment. Oxidative stress markers, such as malondialdehyde (MDA) and hydrogen peroxide, were substantially reduced, while antioxidant enzyme activity was boosted. Certain stress-responsive genes (e.g., *TaADC*, *TaPxPAO*,* TaSAMDC*, *TaSPDS*, *TaSOS1*, *TaNHX1*) were upregulated, highlighting the importance of ionic balance and polyamine metabolism in improved stress tolerance. The application of Cs-Pro and Cs-Gly nanoparticles presents a promising approach to enhance wheat’s salinity tolerance by improving physiological, biochemical, and molecular responses.

**Supplementary Information:**

The online version contains supplementary material available at 10.1038/s41598-025-91496-w.

Salt stress is one of the most critical abiotic factors limiting agricultural productivity, especially in regions where salinity levels are increasing due to factors such as irrigation with saline water and soil degradation^1^. This stress condition severely affects plants by for example, impairing water and nutrient uptake, induction of stomatal closure, inhibition of photosynthesis, induction of oxidative stress and decrease of cell growth and division decreasing biomass, leading to substantial reductions in crop yields^2^. Bread wheat (*Triticum aestivum*L.) is the most widely cultivated cereal crop in the world and serves as a staple food for roughly 35% of the global population. Among cereal crops, wheat exhibits a moderate level of salt tolerance, being less tolerant than barley but more tolerant than rice. Unfortunately, the understanding of the salinity tolerance mechanism in wheat is still limited due to its large genome^3^. Thus, developing strategies to mitigate the impacts of salt stress is of paramount importance^4^. Nanomaterials are utilized to assess their potential in enhancing plant growth and alleviating biotic and abiotic stress effects^5^. Recent advancements in nanotechnology have led to the development of various metallic and non-metallic NPs, each with unique properties that can be harnessed for agricultural applications. For example, ZnO NPs have been demonstrated to have positive effects on pea physiological parameters due to the decreased levels of oxidative stress during salt stress^6^. However, green synthesis, using plant extracts or naturally occurring compounds for nanoparticle production may present a more environmentally friendly option^5^.

Chitosan (Cs), as being nontoxic and biocompatible compound with antibacterial, antioxidant, and chelating characteristics has gained popularity, and it was demonstrated that applied as NP provided protection against abiotic stresses, such as salt stress for instance in bean plants^5^. Chitosan’s structure can easily be modified to enhance the uptake and slow the release of certain plant growth regulators, thus eliciting positive responses in plants under both normal and stressed conditions. However, its effectiveness varies based on concentration, structure, application method, plant type, and growth stage^7,8^. Chitosan nanoparticles (CsNPs) have shown promising results in enhancing plant growth, productivity, and stress resistance partly by stimulating antioxidant and defense systems, regulating osmotic balance, enhancing water and nutrient availability and uptake or inducing changes at metabolite and even at level^9,10^. CsNPs loaded with proline and glycine betaine, as both are well-known for their roles in osmoregulation and stress tolerance can have interesting potential in enhancing plant growth and resilience under salt stress^11,12^.

Investigation regarding NP uptake by salt-stressed plants is a promising direction for future research, and recently several studies have been published on this topic indicating their protective effects at several levels. However, the efficiency of NPs may differ regarding the involved protective compounds, in addition, the outcome of the application may also depend on the plant species or even genotypes. Thus, analysis of NP, salt stress and plant interaction is of great importance. To achieve this, the main objectives of this study were: (i) Evaluating the potential protective role of NPs such as chitosan–proline and chitosan–glycine NPs (Cs–Pro and Cs–Gly NPs) against increasing salt stress in wheat, (ii) The physiological and biochemical changes were compared in winter and spring wheat (*Triticum aestivum* L. cv. Heydari and Sepahan) in the aspect of combined salt stress treatment and nanoparticle application in order to reveal genotype-dependent responses.

## Materials and methods

### Plant material and growing conditions

The experiment was carried out in the research greenhouses of the HUN-REN Centre for Agricultural Research Hungary, Martonvásár (47.3195 N, 18.7888 E), as a factorial experiment using a completely randomized design. Seeds of wheat plants (*Triticum aestivum* L. cv. Heydari, Sepahan) were provided by the Agricultural Research Institute of Razavi Khorasan Province, Mashhad, Iran. Heydaricould be considered as a salt-tolerant winter, Sepahanas a salt-sensitive spring cultivar. For sterilization, seeds were placed in sodium hypochlorite solution (1%) for 5 min and then washed extensively with sterile distilled water. After 4 days, the germinated seeds were planted in pots (5 seeds per pot) of soil mixture and 25/20 °C (day/night) temperatures. After 21 days, at the four-leaf stage, plants were sprayed with chitosan–proline and chitosan–glycine NPs (Cs–Pro, Cs–Gly NPs) at concentrations of (0, 200, and 400 mg L^−1^) for three days then, salt stress was applied for three days at concentrations of (0, 200 and 400 mM NaCl). The volume of applied saline solution to each pot was 250 mL, while the control group was supplied with 250 mL water. Subsequently, the leave samples were collected at day 28, instantly frozen in liquid N_2_ and kept for additional analysis at −80 °C.

### Preparation of chitosan–proline and chitosan–glycine NPs (Cs–Pro, Cs–Gly NPs)

Chitosan (Cs) with 75–85% deacetylation (310–375 kDa molecular weight, CAS Number: 9012-76−4), proline, glycine and tripolyphosphate (TPP, CAS Number: 7758-29−4) were obtained from Sigma-Aldrich Co (St Louis, MO, USA). Deionized water (DIW) was used for this experiment. Cs-NPs were prepared according to Jafari et al.^13^. In summary, a Cs solution was obtained by adding 1 g of Cs powder to 1 L of distilled water and 1000 µL of acetic acid under stirring for 1 h at room temperature. Separately, 0.2 g and 0.4 g of proline and glycine were added to 100 mL of DIW and dissolved by shaking vigorously. The proline and glycine solutions were then added to the Cs solution separately. The ratio of Cs to TPP by weight was 2.5:1, so 0.4 g of TPP was dissolved in 50 mL of DIW and slowly added to the Cs-Pro and Cs-Gly solutions.

### Plant growth parameters, and relative water content (RWC)

The height of plants was measured by a ruler and SPAD meter was used to measure the chlorophyll content in plant leaves. The relative water content (RWC) of leaves in treated and untreated salt-stressed plants was determined by the method of Mullan and Pietragalla^14^. Initially, fresh leaves were weighed for fresh weight (FW), then, turgid weight (TW) was measured after keeping leaf samples in distilled water for 24 h. Finally, the dry weight (DW) was determined after 24 h drying at 70 °C. RWC was determined by the formula:

$$\text {Leaf RWC}{\text{ }}\left( \% \right)=\left( {\left( {\text {FW-DW}} \right)/\left( {\text {TW-DW}} \right)} \right) \times 100$$

### Antioxidant enzymes, malonaldehyde (MDA) and hydrogen peroxide (H2O2) content

Measurements for antioxidant enzyme activities were performed as previously described by Pál et al.^15^. The catalase (CAT) (EC 1.11.1.6) activity was measured spectrophotometrically by monitoring the decrease in absorbance at 240 nm, the glutathione reductase (GR) (EC 1.6.4.2) activity was determined at 412 nm, the guaiacol-peroxidase (GPX) (EC 1.11.1.7) activity was measured at 470 nm, and the glutathione-S-transferase (GST) (EC 2.5.1.18) activity was measured by monitoring the changes at 340 nm with a spectrophotometer (Shimadzu UV-VIS 160 A). Enzyme activities were expressed in nkatal g^−1^ fresh weight (FW). The lipid peroxidation analysis was based on MDA level according to Pál et al.^15^, which was measured spectrophotometrically at 532 nm, with the subtraction of non-specific absorption at 600 nm. For quantification an extinction coefficient of 155 mM^−1^ cm^−1^ was used and values were expressed as nmol g^−1^ fresh weight. The ferrous ammonium sulphate/xylenol orange (FOX-1) method was used to determine the H_2_O_2_content of the samples^16^.

### Proline measurement

The proline (PRO) content was determined on the basis of its reaction with ninhydrin, according to the Bates method^17^.

### Polyamine analysis

The analysis was carried out as described by Németh et al.^18^. The polyamines, namely putrescine (PUT), spermidine (SPD), spermine (SPM), and 1,3-diaminopropane^19^, the terminal oxidation product of SPD and SPM, were analysed as dansylated derivatives via HPLC using a W2690 separation module on a reverse phase column (Kinetex C18, 5 μm, 100 × 2.1 mm, Phenomenex, Torrance, CA, USA.) and a W2475 scanning fluorescence detector with excitation at 340 nm and emission at 515 nm (Waters, Milford, MA, USA).

### Sodium and potassium content in leaf and root

Winter and spring wheat plants were exposed to salinity (0, 200 and 400 mM NaCl) and foliar spraying of Cs-Pro and Cs-Gly NPs (0, 200, and 400 mg L^−1^) for three days before being harvested and air dried at 70 ˚C for 48 h. Potassium and sodium contents were determined from dry leaves and roots after the microwave digestion with a HNO_3_/H_2_O_2_ mixture by the optical emission spectrometry technique (Perkin Elmer Optima 8300 ICP-OES, USA). The measurement was performed by Eurofins Minerág Ltd. (Szekszárd, Hungary).

### Real-Time PCR

Total RNA was extracted from leaf tissues of three independent wheat plants of each treatments using TRIzol reagent according to the manufacturer’s guidelines. RNA samples quantity was determined by NanoDrop (2000c, Thermo Scientific, USA). RNA samples were subjected for DNaseI treatment and removed with a Direct-zol ™ RNA MiniPrep Kit (Zymo Research, Irvine, CA, USA) according to the manufacturer’s instructions. RNA to cDNA was converted using M-MLV reverse transcriptase from (Promega Corporation, Madison, WI, USA). *TaActin* was stable under different conditions, was used as a reference gene for RT-qPCR analysis. PCRBIO SyGreen Mix (PCR Biosystems, London, UK) and CFX96 Real-Time PCR Detection System (Bio-Rad, Hercules, CA, USA) were used for RT-qPCR. Three biological replicates and three technical replicates were performed for each treatment, and the relative expression of selected genes (Table 1) were compared by the 2^−ΔΔCT^method^20^.

**Table 1:** Genes and primers used for RT-qPCR analyses in the present study.

Gene	Forward primer (5'-3')	Reverse primer (5'-3')	Reference
*TaActin*	GTGTACCCTCAGAGGAATAAGG	GTACCACACAATGTCGCTTAGG	^24^
*TaNHX1*	CAGTATGTTGGTATGTTCATGGTC	GATAGAAGCAACAACAAGAGCAG	^25^
*TaSOS1*	CATGCTGGGAGAGTCCACT	ACACGCGGCCTCTGCTCT	^26^
*TaSOS2*	GAAAACCTGCTTCTTGATTCACG	GCTGCAGATCCATCATAGCC	^26^
*TaSOS3*	GTTCGACCTCTTCGATCTCAAG	GAACGTCGTCGTAATGTCCTG	^27^
*TaSAMDC*	ACAGCCTTCTCCACACAAGA	TCCAGACCAGTCATGCACA	^27^
*TaSPDS*	AGGTATTCAAGGGTGGCGTG	TGGGTTCACAGGAGTCAGGA	^27^
*TaADC*	TCTACCCCGTCAAGTGCAAC	GACGAGGCAGCTCATGGT	^27^
*TaPxPAO*	GCTCATAAATCAGCCCAATTCCA	TTCGCCATTTGTTGAGCTCT	^28^

### Promoter cis-element analysis of PA biosynthesis genes in wheat

To gain a deeper understanding of the transcriptional regulation of *PA* genes promoters and their potential function, 1.5 Kb upstream sequence were screened for the presence of *cis*-acting regulatory elements (CREs) from *Triticum aestivum*genome database (IWGSC RefSeqv2.1), then were uploaded to PlantCARE database^21^.

### Statistical analysis

The statistical analysis of data was performed based on the subsequent LSD’s range test at a significance level of *P*≤ 0.05 with four replicates, and two-way ANOVA was applied by SAS software version 9.4^22^. Correlation analyses and principal component analyses (PCA) were assessed to determine the relationships between the traits using SRPLOT^23^. GraphPad Prism (version 9.0.1) was employed to create visual representations of the data associated with Na^+^ and K^+^ contents and gene expression patterns.

## Results and discussion

### Effect of salt, Cs-Pro and Cs-Gly NPs on RWC, shoot height, and proline, MDA and H2O2 contents

According to the results presented in Table 2., the highest RWC (86%) was obtained in the Cs-Gly NPs (400 mg L^−1^) Heydari plants under control conditions and the lowest RWC (28%) was detected in Sepahan cultivar, when plants were subjected to salt stress (400 mM NaCl) and Cs-Pro NPs (400 mg L^−1^). The results indicated that 200 and 400 mg L^−1^Cs-Pro and 400 mg L^−1^ Cs-Gly NPs efficiently alleviated stress in Heydari, but not in Sepahan. In addition, the treatments with NPs decreased the RWC values even without salt stress conditions in Heydari. It was confirmed, that Sepahan cultivar was more sensitive to salt than Heydari, which was also manifested in differences in other stress markers (Table 2). Increasing salt stress generally reduces plant growth, as indicated by lower plant height. Under the present conditions, salt stress-induced plant growth was also observed, in addition, as it was expected the tolerant one, Heydari performed better under salt stress compared to Sepahan. Cs-Pro treatments could hardly influence the plant height values regardless of the salt concentration in either genotype, while 400 mg L^−1^ Cs-Gly had a positive effect on shoot weight both at lower and higher salt stress conditions again only in Heydari (Table 2).

Visual symptoms also showed that high salinity caused more severe chlorosis in Sepahan than in Heydari (Fig. S1), and SPAD measurement also confirmed this at higher salt concentration (Table 2). Interestingly, NPs treatment in certain cases provided protection against salt stress-induced decrease of chlorophyll content, namely Cs-Pro at 400 mg L^−1^ at 200 and 400 mM salt stress in both genotypes, and Cs-Gly especially when applied in 400 mg L-^1^ concentration under both salt stress conditions, and in both genotypes (Table 2).

Salt stress may also increase the MDA and H_2_O_2_ levels as markers of oxidative stress level. Under the present conditions, salt treatments alone caused a slight but statistically significant increase in the MDA levels in both genotypes. Interestingly, similar level of increase was also found after the foliar application of NPs. MDA content decreased only in the combination of 200 mg L^−1^ Cs-Gly or Cs-Pro with 200 mM NaCl in the case of Heydari, and in the combination of 400 mg L^−1^ Cs-Pro with 200 mM NaCl in the case of Sepahan under salt stress conditions (Table 2).

The H_2_O_2_ levels were substantially affected by salt stress only in Heydari (Table 2). As in most cases NP treatments alone also increased its level in both genotypes, NP treatment-induced decreases in the levels of H_2_O_2_ were only observed in case of Heydari at 200 mM NaCl. These results support the view that H_2_O_2_ levels do not directly reflect the physiological status of plants, but that H_2_O_2_plays a complex role in the regulation of acclimation processes to unfavourable conditions, especially if it is associated with oxidative stress^29^.

PRO protects cells by enhancing osmotic regulation, inhibiting the accumulation of reactive oxygen species (ROS), and maintaining membrane structure^30^. Proline also serves as an osmotic stabilizer and protects enzymes, proteins, and membranes^31^. Increased proline levels may indicate reduced stress level^11^. Salinity increased the PRO content compared to controls in a concentration-dependent mode only in the sensitive genotype. Data presented in Table 1 showed that the PRO level in Sepahan was already higher in Heydari plants under control conditions, and this difference was also detected after 200 or 400 mM salt treatments. Cs-Pro treatments induced PRO accumulation in both genotypes, which was concentration-dependent under salt stress conditions indicating its role in better osmotic adjustment. It can be also observed that in the tolerant genotype, higher fold changes were induced by the combined treatments of Cs-Pro and salt compared to the sensitive one.

**Table 2:** The impact of foliar spraying of Cs–Pro and Cs–Gly NPs (0, 200, and 400 Mg L^−1^) on morpho-physiological properties of two wheat cultivars (Heydari, Sepahan) under salt stress (0, 200 and 400 mM NaCl). In each column, values followed by the same letter(s) do not have significant difference at α = 0.05. Distilled water was used as a control.

Salt	Cultivar	Treatments	RWC (%)	Height (cm)	SPAD	MDA (nmol g-1 FW)	H_2_O_2_ (µM g-1 FW)	PRO (nmol g-1 FW)
0	Heydari	Control	81.06^d^	38.31^b^	28.22^gh^	6.53^ef^	21.30^jk^	66.17^nop^
		Cs–Pro 200 mg L^−1^	69.14^fg^	40.55^a^	28.65^gh^	8.50^ab^	31.30^efg^	167.05^ij^
		Cs–Pro 400 mg L^−1^	80.50^de^	35.25^dce^	28.63^gh^	6.83^ef^	27.06^ghi^	145.02^kj^
		Cs–Gly 200 mg L^−1^	70.01^fg^	38.25^b^	39.83^a^	7.73^cd^	31.91^efg^	79.24^n^
		Cs–Gly 400 mg L^−1^	86.28^a^	41^a^	31.36^cd^	7.69^cd^	24.14^ijk^	76.99^no^
	Sepahan	Control	60.80^ij^	34^ef^	28.35^ghi^	5.87^g^	10.59^l^	108.49^lm^
		Cs–Pro 200 mg L^−1^	46.71^lm^	37^bc^	29.70^ef^	7.97^cd^	31.86^efg^	346.08^de^
		Cs–Pro 400 mg L^−1^	50.84^kl^	36.5^dc^	32.75^bc^	7.74^cd^	20.12^k^	358.72^d^
		Cs–Gly 200 mg L^−1^	51.20^kl^	38^b^	30.92^de^	7.13^cd^	25.75^jhi^	74.69^no^
		Cs–Gly 400 mg L^−1^	43.49^lmn^	36.50^dc^	29.12^ef^	6.83^ef^	10.51^l^	171.86^i^
200 mM	Heydari	Control	68.57^gh^	36.25^dc^	27.11^gh^	8.13^ab^	57.19^b^	37.82^q^
		Cs–Pro 200 mg L^−1^	83.36^bc^	35^dce^	27.82^ij^	7.75^cd^	35.29^de^	260.35^g^
		Cs–Pro 400 mg L^−1^	76.67^ef^	33.25^fg^	34.63^b^	8.50^ab^	23.08^ijk^	299.97^f^
		Cs–Gly 200 mg L^−1^	68.08^gh^	34^ef^	31.70^cd^	6.40^ef^	38.25^dc^	52.21^qop^
		Cs–Gly 400 mg L^−1^	81.77^d^	40^a^	29.16^ef^	8.81^ab^	33.71^def^	45.63^qp^
	Sepahan	Control	80.48^de^	32^ij^	27.50^ij^	7.70^cd^	11.37^l^	144.46^kj^
		Cs–Pro 200 mg L^−1^	62.20^hij^	34^ef^	27^ijk^	7.41^cd^	13.76^l^	355.85^de^
		Cs–Pro 400 mg L^−1^	41.62^lmn^	34^ef^	32.47^bc^	6.30^ef^	12.48^l^	436.80^c^
		Cs–Gly 200 mg L^−1^	40.53^mn^	36^dc^	31.85^cd^	8.93^ab^	74.48^a^	122.45^kl^
		Cs–Gly 400 mg L^−1^	45.51^lm^	33.5^fg^	25.85^lm^	9.12^b^	24.12^ijk^	83.91^nm^
400 mM	Heydari	Control	64.53^hi^	31^j^	25.10^lm^	7.46^cd^	30.87^efg^	69.02^nop^
		Cs–Pro 200 mg L^−1^	84.79^c^	32.25^hi^	27.43^ijk^	9.68^b^	32.67^ef^	226.86^h^
		Cs–Pro 400 mg L^−1^	77.66^ef^	31^j^	27.30^ijk^	8.51^ab^	29.41^fgh^	332.06^e^
		Cs–Gly 200 mg L^−1^	58.72^ijk^	33^fgh^	26.90^kl^	10.33^a^	34.94^de^	68.25^nop^
		Cs–Gly 400 mg L^−1^	85.51^b^	33^fgh^	30.80^de^	7.79^cd^	41.27^c^	82.46^n^
	Sepahan	Control	61.56^hij^	29^k^	20.80^p^	7.02^cd^	13.19^l^	231.97^h^
		Cs–Pro 200 mg L^−1^	63.44^hi^	32^ij^	21.42^o^	7.85^cd^	21.58^jk^	549.10^b^
		Cs–Pro 400 mg L^−1^	28.42^p^	31^j^	26.40^kl^	8.34^ab^	14.70^l^	649.13^a^
		Cs–Gly 200 mg L^−1^	39.36^o^	31.25^j^	25.50^lm^	7.78^cd^	13.46^l^	130.22^kl^
		Cs–Gly 400 mg L^−1^	41.63^mn^	31^j^	24.26^mn^	8.28^ab^	14.77^l^	226.27^h^

RWC = relative water content; MDA = malondialdehyde; H_2_O_2_ = hydrogen peroxide; PRO = proline.

Results of statistical analysis showed that salt stress (S), cultivar (C), and nanoparticle treatments (NT) have significant effects on morphological and biochemical properties, RWC and antioxidant enzyme activities (Table S1). Interactions between these factors (S × C, S × NT, C × NT, S × C × NT) are also significant for many parameters, suggesting that the response to salt stress and treatment varies between the cultivars and indicating complex interactions between the factors. For example, salt stress has a highly significant effect on all the parameters. NT has a highly significant impact on all parameters except MDA, where it is not significant. Similarly to our research, an increased lipid degradation rate and MDA production together with increased proline levels, resulting in ROS formation that caused cell damage under salt stress conditions were noted in numerous studies^32,33^. Application of certain protective compounds, for example, salicylic acid, proline or glycine betaine have been demonstrated to decrease MDA levels and reduce stress symptoms during salinity stress in plants^34–36^. In another study, it was noted that SiO2 NPs significantly reduced the negative impacts of drought stress by increasing photosynthesis and RWC^37^. Additionally, chitosan pre-treatment under salinity stress also lowered MDA levels and enhanced antioxidant enzyme activities, mitigating the adverse effects of salinity^38^.

All the above-mentioned results indicate that NPs treatments may induce mild stress processes in wheat plants, leading to a slight elevation in MDA and H_2_O_2_ levels. However, these treatments may provide protection against a subsequent stress. Based on the results on RWC, shoot height and SPAD parameters, in addition to MDA and H_2_O_2_ contents, it can be concluded that more protective effects of NPs were observed in the tolerant genotype, especially under lower salt concentration. A particularly positive effect of 400 mg L^−1^ Cs-Pro NP was found almost in all measured parameters of Heydari, and only in certain parameters in the case of the sensitive genotype. In the next parts, the molecular mechanisms induced by the interactions of salt and NPs treatments were investigated further.

### Effects of Cs–Pro and Cs–Gly NPs on root and leaf K⁺ and na⁺ content under salt stress

**Fig. 1:**
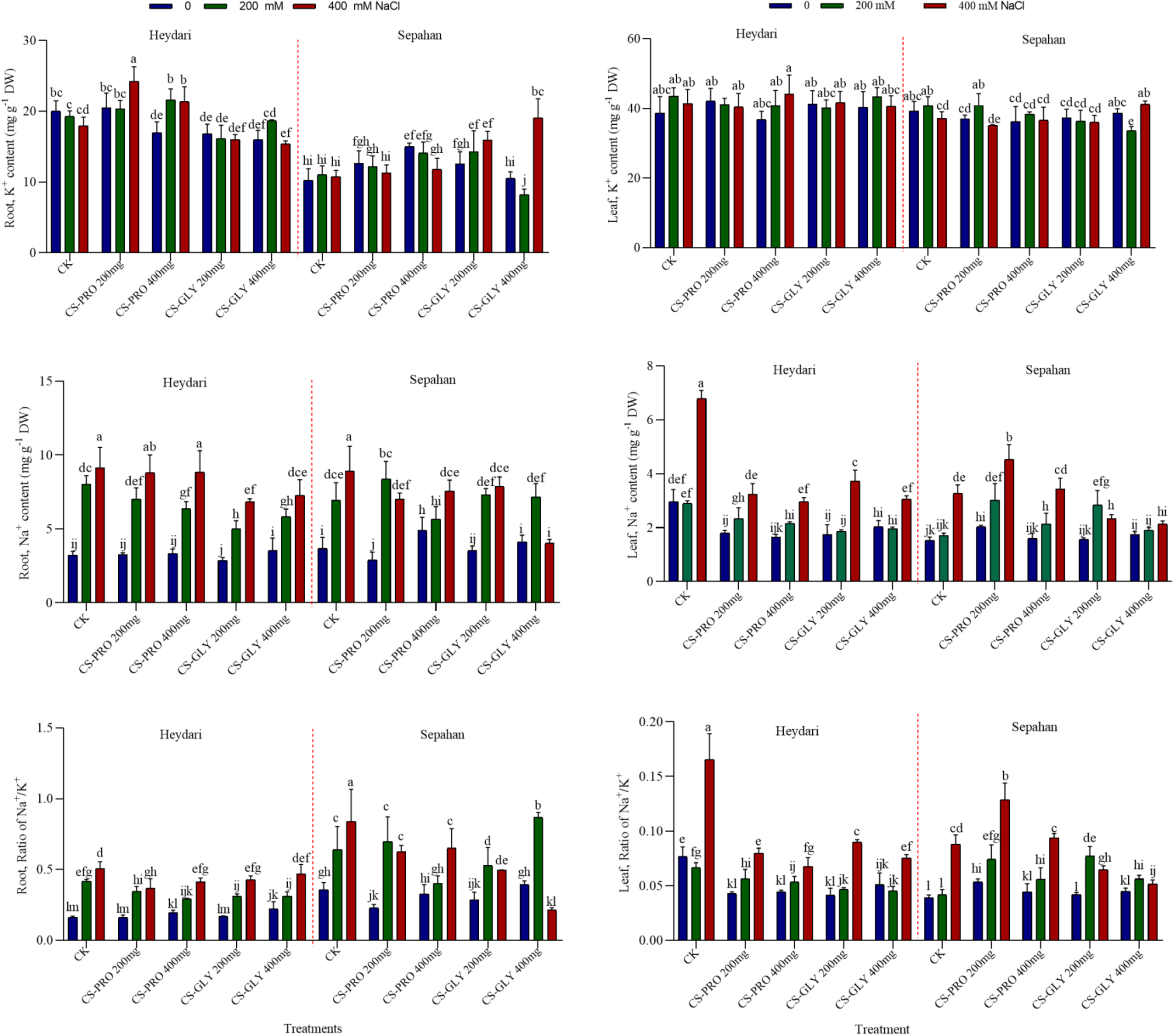
Na^+^ and K^+^ contents, and the Na^+^/K^+^ ratios in leaf and root samples of two wheat cultivars (Heydari, Sepahan) under salt stress. Different letters indicate significant differences after mean comparisons by LSD test at *p <* 0.05.

As salt concentration increases (200 and 400 mM NaCl), the Na⁺ content generally increased in most of treatments. This increase was usually significant in the roots at both 200 and 400 mM, but only at 400 mM in the leaves. The most substantial increase in the leaf Na^+^ content was detected at 400 mM salt stress in Heydari (Fig. 1). Cs-Pro and Cs-Gly NPs appear to reduce Na⁺ content compared to the control under salt stress in Heydari (Fig. 1). Cs-Gly (200 mg L^−1^) in root and leaf reduce Na⁺ content in Heydari compared to controls. Potassium plays a key role in cellular stress tolerance, regulating transpiration, water uptake, and CO_2_supply for photosynthesis through stomatal opening, cell expansion, and osmoregulation during salt stress^39^. K⁺ content remains relatively stable across treatments, with some increase under higher nanoparticle concentrations, even under salt stress in Heydari, but it is lower in Sepahan especially under salt stress in root (Fig. 1). Potassium uptake in roots is closely related to sodium levels; excess sodium from salt stress may reduce potassium absorption. Consistent with our findings, both glycine betaine and proline treatments were efficient against salt stress due to the modified Na^+^uptake^34,40^.

In addition, Gong et al.^41^, showed that Cs-Se NPs complexed with Na⁺ at the root level and reduced Na⁺ absorption by decreasing apoplastic transport to the leaves, and exerted a positive effect.

The salt stress levels have a highly significant effect on Na⁺ content and Na⁺/K⁺ ratio in both leaf and root, and K⁺ content in the root in two wheat cultivars. However, K⁺ content in the leaf is not significantly affected by salt stress (Table S2). The NT had a significant impact on all parameters except for K⁺ content in the leaf, indicating that these treatments can alter Na⁺ and K⁺ content, especially under salt stress conditions (Table S2).

The results indicate that S, C, and NT have significant effects on Na⁺ and K⁺ content in the leaves and roots of wheat plants. The interactions between these factors are also significant, suggesting that the responses to salt stress and nanoparticle treatments are complex and depend on the specific combination of S, C, and NT. Cs-Pro and Cs-Gly NPs mitigate the adverse effects of salt stress, particularly by reducing Na⁺ accumulation and maintaining K⁺ levels in the leaves.

This effect was more pronounced in the tolerant cultivar compared to the sensitive one indicating better management of ion balance under salt stress. On the other hand, Na^+^ accumulation in the root was higher in both cultivars than in the leaves, which indicates its greater sensitivity to salt stress. These findings suggest that chitosan nanoparticles with various active components can be valuable in developing strategies to enhance salt tolerance in wheat, improving plant growth and productivity under saline conditions.

### Effects of salt, Cs-Pro and Cs-Gly NPs on antioxidant enzymes activities

Under high saline conditions, plants may elevate ROS levels, which oxidize lipids, proteins, and other cellular components, resulting in functional loss, but they also play as secondary signaling molecules to induce stress responses. Successful plant growth and adaptation largely rely on the ability to regulate the ROS levels by increase antioxidant compounds like ascorbate and glutathione, and to increase the activities of the antioxidant enzymes, to mitigate oxidative stress^42^. In many plants, salinity also triggers the increased activity of antioxidant enzymes and the accumulation of non-enzymatic antioxidants, which means an efficient strategy for plants to defend against oxidative stress and adapt to salt stress^43^.

In the present case, four detoxification enzymes, namely GR, GST, GPX, and CAT were investigated. In control, untreated plants, GR had higher, GST, GPX, and CAT had lower activities in Heydari than in the Sepahan variety. GR is essential in the ascorbate-glutathione cycle and acts as a key scavenger of H_2_O_2_in cells. This oxido-reductase enzyme converts oxidized glutathione (GSSG) to reduced glutathione (GSH) using NADPH as a cofactor. To withstand climatic and environmental stresses, plants must maintain a high GSH: GSSG ratio, as GSH is a powerful antioxidant that removes ROS from the cell^43^. However, under the present environmental conditions, salt stress did not induce the GR activities in the used wheat genotypes, the application of all the NPs treatments led to elevated GR levels in Sepahan, and in many cases in Heydari, especially at 400 mM NaCl (Table 3). Although the highest GST activity was detected in Sepahan at 400 mM NaCl concentration in the Cs- Gly 200 mg L^−1^ treated plants, in general, the highest induction by NPs was observed in the case of CS-Pro 400 mg L^−1^ treatment, except in Heydari at 400 mM salt. Nevertheless, salt-induced GST increase could not be observed in the plants, which were not treated with NPs. In contrast to GST, the CAT activity showed a significant increase in plants treated with high salinity (400 mM), so the utmost activity was shown in Sepahan at 400 mM NaCl of Cs- Pro 400 mg L^−1^ and the lowest in Heydari under control condition (Table 3). While salt treatment caused a concentration-dependent increase in Heydari GPX, it caused a significant decrease in Sepahan. The highest GPX was observed at 400 mM NaCl in Sepahan of Cs-Pro 200 mg L^−1^, and the lowest at 200 mM NaCl and Cs-Gly 200 mg L^−1^ (Table 2). Cs-Pro 200 mg L^−1^ also substantially increased the GPX activity at 200 mM salt. Zafar et al.^44^found a significant increase in antioxidant enzymes, particularly GR, in black mustard seedlings treated with zinc NPs. Similar results were reported in other studies^45,46^. In both cultivars, enzyme activities generally increase with treatments (Cs-Pro and Cs-Gly), indicating a stress response mechanism under salt stress. However, the effects of NPs on the CAT activities were not uniform. The results indicate that the regulation of antioxidant enzyme activities is complex, influenced by salt stress, various cultivars, and the NP treatments applied (Table S1).

**Fig. 2:**
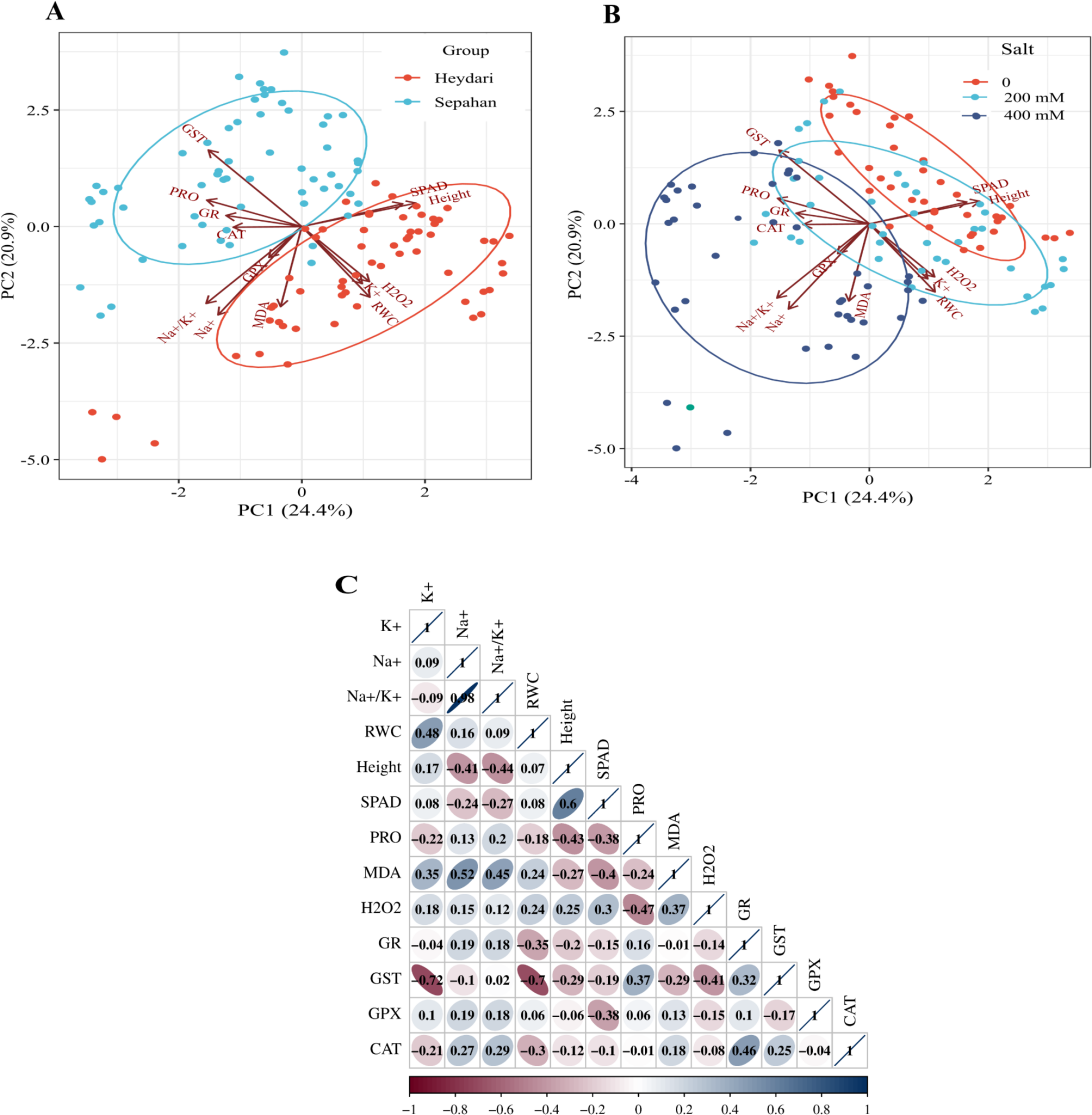
Principal component analysis for the effect of foliar spraying of nanocomposites on morpho-physiological properties of two wheat cultivars (Heydari, Sepahan) (A), under salt stress (0, 200 and 400 mM NaCl) (B), Pearson correlation coefficients (C). RWC = relative water content; PRO = proline; MDA = malondialdehyde; H_2_O_2_ = hydrogen peroxide; GR = glutathione reductase; GST = glutathione-S-transferase; GPX = guaiacol-peroxidase; CAT = catalase. *, **, ***: significant at 5%, 1%, and 0.1% probability levels, respectively.

In a recent study also on wheat, where 20 genotypes with different salt tolerance levels were compared, it was concluded that tolerant genotypes might have a more active antioxidant system, as in general higher antioxidant enzyme activities were observed in tolerant moderately tolerant genotypes than in sensitive cultivars. However other processes such as the regulation of Na^+^uptake or osmotic balance as part of the complex adaptation are also important parts of the tolerance mechanism^47^. In order to reveal more relationships between the results on the investigated parameters pearson correlation and principal component analysis were also performed. Pearson’s correlations of height, SPAD, RWC, PRO, MDA, H_2_O_2_, Na^+^, K^+^, Na^+^/K^+^, CAT, GPX, GR and GST are exhibited in (Fig. 2C). K⁺ is positively correlated with RWC, indicating relationship between better water retention and K^+^ content. While Na⁺ and Na⁺/K⁺ ratio and PRO content are negatively correlated with height and SPAD values, suggesting that higher sodium levels or an unfavourable Na⁺/K⁺ balance negatively affect wheat growth and photosynthesis. While oxidative stress markers such as MDA content was in positive correlation with Na^+^ and Na^+^/K^+^ ratio, and in a negative correlation with height, SPDA and PRO values. These relations showed that higher levels of oxidative damage correspond with reduced growth and water retention underscoring the detrimental effects of salt stress in wheat. Antioxidant enzymes (GPX and CAT) showed positive correlations with MDA, this indicates that the activity of these enzymes also increases as a defensive response (Fig. 2C).

Individual PCA plots were constructed for wheat plants subjected to 0, 200, and 400 mM NaCl salt stress and treated with 0, 200, and 400 mg L^−1^ Cs-Pro and Cs-Gly in two wheat cultivars (Heydari and Sepahan). The analysis displayed the 15 best-fitting variables (height, SPAD, RWC, PRO, MDA, H_2_O_2_, Na^+^, K^+^, Na^+^/K^+^, CAT, GPX, GR and GST) (Fig. 2A, B). PCA bi-plots of treatment variable associations displayed lines from the center, indicating positive or negative correlations among various variables. The PCA showed that both salt concentration and cultivars play critical roles in determining the plant’s physiological responses, as indicated by the spread and clustering of data points. Na⁺, Na⁺/K⁺ ratio, and antioxidant enzyme activities significantly influence the variation observed, particularly at higher salt concentrations, where Na⁺ and Na⁺/K⁺ ratio are positively correlated with increased salinity, while RWC are negatively correlated, highlighting their association with stress response (Fig. 2A, B).

**Table 3:** The impact of foliar spraying of Cs-Pro and Cs-Gly NPs (0, 200, and 400 Mg L^−1^) on antioxidant enzyme activities of two wheat cultivars (Heydari, Sepahan) under salt stress (0, 200 and 400 mM NaCl). In each column, values followed by the same letter(s) do not have significant difference at α = 0.05. Distilled water was used as a control.

Salt	Cultivar	Treatments	GR (nkatal/g FW)	GST (nkatal/g FW)	GPX (nkatal/g FW)	CAT (nkatal/g FW)
0	Heydari	Control	19.46^ghi^	20.78^hi^	615.75^jkl^	13,732^j^
		Cs–Pro 200 mg L^−1^	24.55^ef^	13.89^klm^	1011^dc^	20,412^efg^
		Cs–Pro 400 mg L^−1^	19.18^ghi^	29.97^def^	1076^dc^	15,682^hij^
		Cs–Gly 200 mg L^−1^	19.20^ghi^	17.86^jkl^	1199^b^	26,806^bcd^
		Cs–Gly 400 mg L^−1^	22.24^gh^	19.15^hij^	629.47^jkl^	16,685^hij^
	Sepahan	Control	15.19^k^	30.88^de^	1087^bc^	20,757^ef^
		Cs–Pro 200 mg L^−1^	27.81^abc^	31.41^d^	745.96^ghi^	24,168^d^
		Cs–Pro 400 mg L^−1^	27.80^abc^	41.73^ab^	1208^b^	17,345^ghi^
		Cs–Gly 200 mg L^−1^	23.52^efg^	38.42^bc^	697.14^ijk^	25,745^dc^
		Cs–Gly 400 mg L^−1^	27.20^abc^	40.31^ab^	506.87^lmn^	18,520^gh^
200 mM	Heydari	Control	18.23^hi^	12.84^lm^	780.75^gh^	15,366^ij^
		Cs–Pro 200 mg L^−1^	18.76^hi^	18.23^ijk^	877.89^efg^	18,405^gh^
		Cs–Pro 400 mg L^−1^	24.13^ef^	21.58^ghi^	928.82^de^	17,345^ghi^
		Cs–Gly 200 mg L^−1^	23.71^efg^	13.39^klm^	741.35^hij^	17,631^ghi^
		Cs–Gly 400 mg L^−1^	16.17^ijk^	15.00^kl^	905.86^def^	18,549^gh^
	Sepahan	Control	17.94^ij^	30.97^de^	348.77^o^	16,399^hij^
		Cs–Pro 200 mg L^−1^	29.22^ab^	26.37^efg^	1376^ab^	25,401^dc^
		Cs–Pro 400 mg L^−1^	24.07^ef^	37.96^bc^	591.88^klm^	20,785^e^
		Cs–Gly 200 mg L^−1^	27.97^abc^	39.22^abc^	469.50^mno^	20,756^ef^
		Cs–Gly 400 mg L^−1^	22.46^gh^	34.77^dc^	925.71^de^	27,436^abc^
400 mM	Heydari	Control	19.20^ghi^	23.05^gh^	1083 ^bc^	29,529^ab^
		Cs–Pro 200 mg L^−1^	25.59^cde^	26.04^fg^	916.09^de^	17,488^ghi^
		Cs–Pro 400 mg L^−1^	22.00^gh^	19.76^hij^	839.10^efg^	18,606^gh^
		Cs–Gly 200 mg L^−1^	30.28^a^	18.41^ijk^	1113 ^bc^	20,155^efg^
		Cs–Gly 400 mg L^−1^	26.81^cd^	23.08^gh^	829.17^efg^	20,642^ef^
	Sepahan	Control	19.29^ghi^	31.88^d^	414.74^no^	27,752^abc^
		Cs–Pro 200 mg L^−1^	26.57^cd^	31.48^d^	1473^a^	15,732^hij^
		Cs–Pro 400 mg L^−1^	28.37^ab^	40.28^ab^	947.07^de^	30,418^a^
		Cs–Gly 200 mg L^−1^	25.38^cde^	43.82^a^	644.81^jk^	20,154^efg^
		Cs–Gly 400 mg L^−1^	25.98^cde^	31.18^de^	1095^bc^	16,829^hij^

GR = glutathione reductase; GST = glutathione-S-transferase; GPX = guaiacol-peroxidase; CAT = catalase.

### Effects of salt, Cs–Pro and Cs–Gly NPs on salt tolerance-related gene expression

**Fig. 3:**
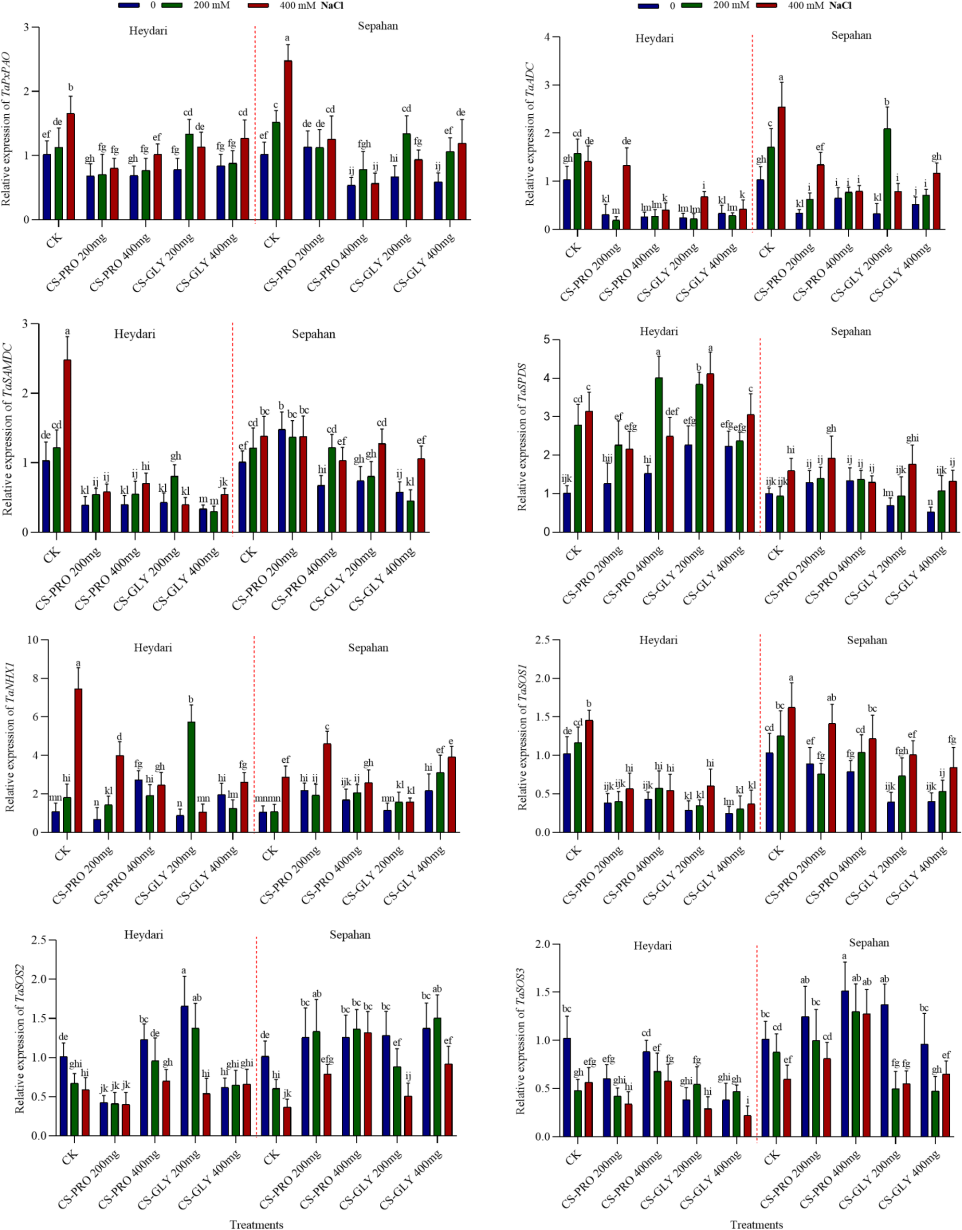
The effect of Cs-Pro and Cs-Gly NPs (0, 200, and 400 mg L^−1^) on the expression patterns of two wheat cultivars (Heydari, Sepahan) under salt stress (0, 200 and 400 mM NaCl). *PxPAO* (Peroxisomal polyamine oxidase); *ADC* (arginine decarboxylase); *SAMDC* (S-adenosylmethionine decarboxylase); *SPDS* (spermidine synthase); *NHX* (vacuolar Na^+^/H^+^ antiporter); *SOS* (salt overly sensitive) genes. Each column is the mean expression of 3 technical and 3 biological replicates. Error bars are standard deviations of biological replicates. Lower-case letters above bars indicate mean comparisons from LSD test at *p <* 0.05.

The vacuolar Na^+^/H^+^ antiporter *NHX* gene may play a substantial role in the development of salt tolerance in plants. Overexpression of *AtNHX1*could dramatically increase the salt tolerance of tomato plants, which was able to set fruit even at 200 mM NaCl^48^. Jiang et al.^49^, found that overexpressing the *NHX1* gene in poplar enhances Na^+^ ion sequestration in vacuoles and improves salt stress resistance. Transgenic *Arabidopsis* plants also achieved salt tolerance through the Na^+^/H^+^ antiporter *TaNHX1*^50^. In this study, the effects of Cs-Pro and Cs-Gly-NPs on the expression levels of genes involved in PA biosynthesis and metabolism, namely *TaADC*, *TaSAMDC*, *TaSPDS*, *TaPxPAO* were also determined in leaves with or without salt stress. Induction of the genes playing a role in PA biosynthesis could be observed in both cultivars under high saline conditions (Fig. 3). However, NPs treatment substantially reduced the *TaADC*, *TaSAMDC*, and slightly the *TaPxPAO* expression rates, especially in Heydari cultivar (Fig. 3).

Under the present experimental conditions, high salinity generally led to higher expression levels of the *TaNHX1* gene in many cases (Fig. 3). However, the NP-induced *NHX1* expression was not obvious, although in certain cases, for example, CS-Gly-200 mM at 200 mM NaCl, but not at 400 mM NaCl especially in Heydari, it was substantially higher than in the other cases. SOS1, a plasma membrane-type Na^+^/H^+^antiporter, is another component playing a decisive role in plant salt tolerance^51^. In *Arabidopsis*, the mutation in the *SOS1* gene leds to a more dramatic decrease in the salt tolerance, than in *SOS2* or *SOS3*^52^. We also examined the impacts of Cs-Pro and Cs-Gly-NPs on the expression of *TaSOS1*, *TaSOS2*, and *TaSOS3* encoding protective proteins. SOS3 acts as a Ca^2+^sensor, facilitating the movement of SOS2, leading to the release of sodium ions from the cells^35,51^. The expression of the *TaSOS1* gene increased under high salinity in a concentration-dependent way; however, it was generally lower in NPs-treated plants, especially in Heydari (Fig. 3). In contrast to *SOS1*, the expression of the *TaSOS2* and *TaSOS3* genes usually decreased in the leaves of the salt-exposed plants (Fig. 3). NPs also affected the *TaSOS2* and *TaSOS3* expressions, but it dependent on both the concentration and the type of the NPs. While CS-PRO 200 mg reduced the expression of *TaSOS2* at all the salt concentrations in Heydari, but increased in Sepahan; CS-GLY 200 mg significantly increased it at 0 and 200 mM NaCl treatments in both genotypes (Fig. 3). Interestingly, *TaSOS3* expression was generally higher in Sepahan than in Heydari, especially at NPs treatments (Fig. 3).

These results, including the PA levels and the related gene expression values, confirm the view, that the PA metabolism is under a complex regulation mechanism, where both the synthesis and degradation are finely regulated^36^. Nevertheless, the changes during the salt and NPs treatments were not always uniform, indicating a complex regulatory mechanism, keeping the PA levels at the necessary levels. Overall, although the pattern of the *TaNHX1* expression was very similar to that of leaf Na^+^ content (Fig. 1), and the gene expression and Na content values showed a strong (0.7) correlation, it cannot be stated that the stress-mitigating effect of the used NPs is mainly due to the Na^+^/H^+^ antiport process.

### Effects of salt, Cs–Pro and Cs–Gly NPs on PA

**Fig. 4:**
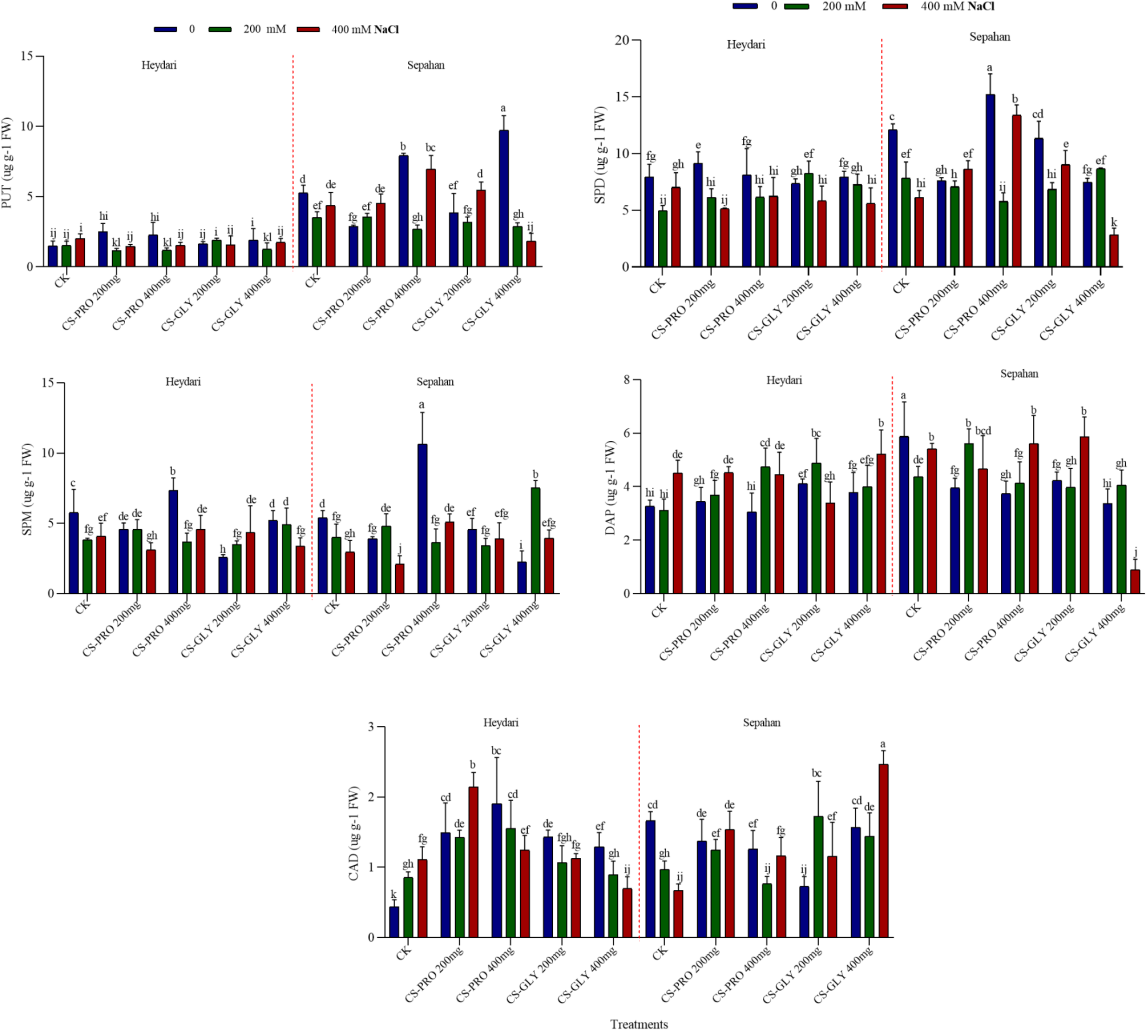
The effect of Cs-Pro and Cs-Gly NPs (0, 200, and 400 mg L^−1^) on PA of two wheat cultivars (Heydari, Sepahan) under salt stress (0, 200 and 400 mM NaCl).

In the present work, three major PAs, namely, PUT, SPD, and SPM, a degradation product, DAP, and another PA, CAD, which is synthesized independently from the PUT-SPD-SPM pathway, were measured in the leaves. Under control conditions, SPD was the most abundant PA in both cultivars. While SPM level was in the same range, PUT was significantly, more than two times, higher in Sepahan than in Heydari (Fig. 4). In control plants, exposure to high salinity generally reduced the SPD and SPM levels. However, CAD increased in Heydari and decreased in Sepahan cultivar. NPs treatments substantially modified the PA levels in both genotypes. For example, CS-Pro 400 mg L⁻¹ significantly increased the PUT, SPD, and SPM levels, parallel with a decrease in DAP in Sepahan, but not in Heydari cultivar (Fig. 4).

### *Cis*‑element analysis of the PA biosynthesis genes in wheat

**Fig. 5:**
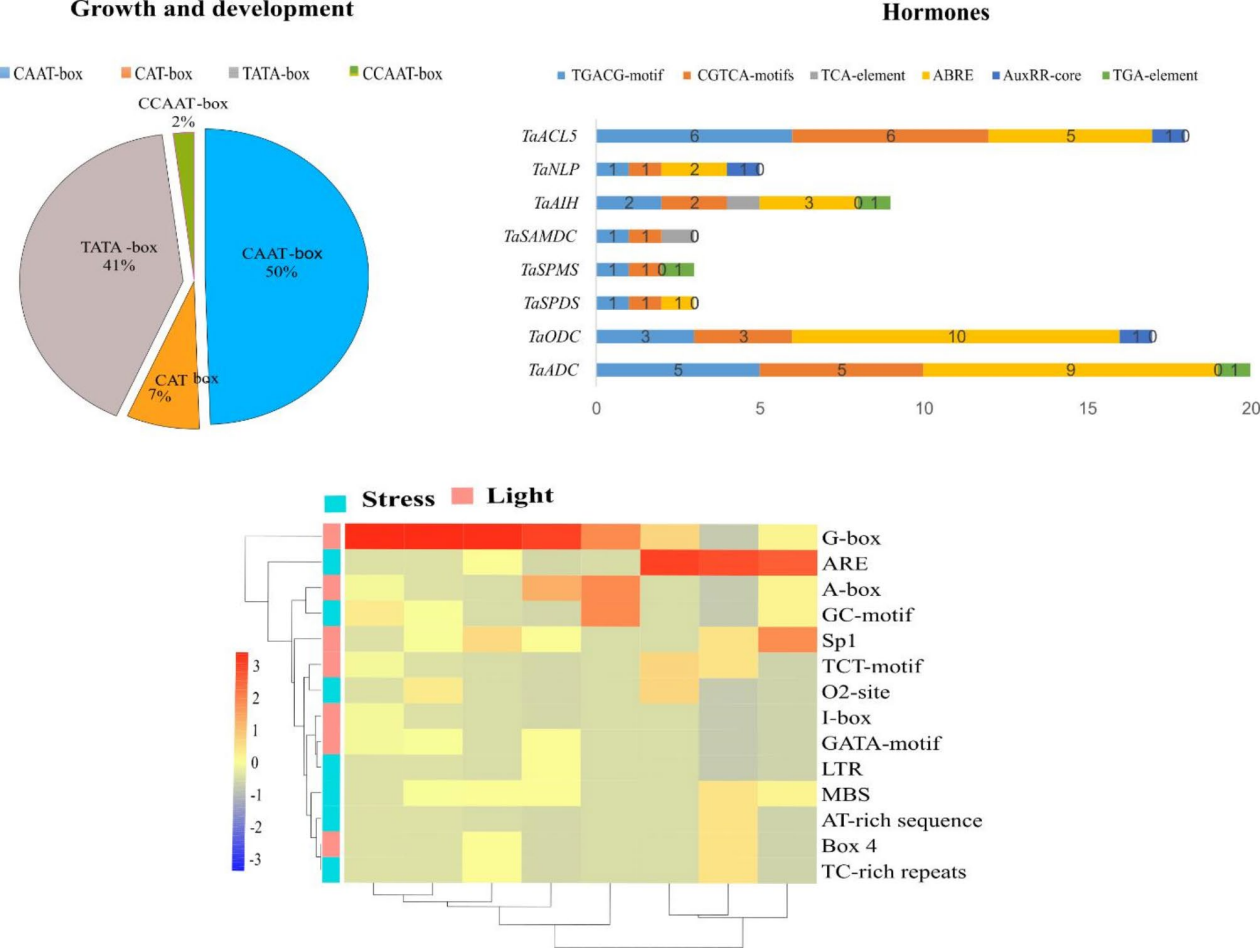
*Cis*-acting regulatory elements identified in the 1.5 kb upstream region of the wheat *PA* biosynthesis genes. The group of elements involved in growth and development, hormone, stress, and light are presented in different colors.

The promoter sequences of *TaPAO* genes were identified in wheat genome by PlantCARE database. The analysis primarily focused on *cis*-elements linked with growth and development, hormone, stress, and light for *TaPAO* biosynthesis genes (Fig. 5). Comparatively, six types of elements were related to hormone response, mainly ABRE involved in abscisic acid (ABA) response, CGTCA-motif and TGACG-motif in jasmonic acid (MeJA) response, TGA-element and AuxRR-core in auxin response, and TCA-element in salicylic acid response (Fig. 5). Additionally, stress responsive elements (7 types) comprised LTR (low temperature response), MBS (drought inducibility), TC-rich repeats (defense stress responsiveness), GC-motif (anoxic specific), O_2_- site in zein metabolism regulation and ARE for anaerobic induction (Fig. 5). The *cis*-acting elements are functioning in response to growth and development such as CAAT-box (50%), TATA-box (41%), CAT-box (7%), and CCAAT-box (2%). The *cis*-acting elements are functioning in response to light including (G-box, A-box, I-box, Sp1, Box4, TCT-motif and GATA-motif). The overall frequency of these hormone-responsive elements was 78 and predominant promoter elements were ABRE, TGACG-motif, and CGTCA-motifs, with frequency of 10, 6, and 6, respectively (Fig. 5). The genes *TaODC*, *TaADC*, and *TaACL5*, respectively, showed the highest levels of ABRE and MeJA hormones, which indicates that these genes can play important role in abscisic acid and methyl jasmonate signaling pathways. Abscisic acid and methyl jasmonate hormones and stress response elements such as ARE for anaerobic induction were in the majority (Fig. 5). ABRE is a crucial *cis*-acting regulatory element for anaerobic induction. Another study revealed that wheat lines with the TaHKT9-BDel-1077 allele, featuring a single auxin-responsive element (ARE) motif in their promoter, showed lower TaHKT9-B mRNA levels and improved salt tolerance, emphasizing the importance of identifying this regulatory element^53^. This study, consistent with Shokri-Gharelo et al.^54^, found that ABRE occurs frequently among *cis*-acting elements related to abiotic factors. *Cis*-elements are essential for modulating gene expression, which regulates physiological processes vital for growth, development, and adaptation to environmental changes^55^. Research suggests that the jasmonic acid signaling pathway plays a role in regulating a plant’s response to salt stress^56^. In a previous study, we identified *cis*-elements related to hormonal and stress responses in the *HKT* gene family of the wheat variety TAM107 under drought, heat, and their combination, finding that ABRE and MeJA elements were predominant, highlighting their crucial role in stress response.

## Conclusions

The application of Cs-Pro and Cs-Gly nanoparticles demonstrated significant potential in mitigating the adverse effects of salt stress on wheat (*Triticum aestivum* L.). Overall, the Cs-Pro 400 mg L⁻¹ treatment emerged as the most effective, enhancing growth and stress resilience across multiple metrics. In addition, the salt-tolerant Heydari cultivar consistently outperformed the sensitive Sepahan cultivar in stress mitigation after NP treatments. These NPs exerted their effects in a very complex manner and at different levels, as influencing plant physiology, metabolism and even gene expression. Salt stress alleviation in varying degrees was manifested in enhanced growth and water homeostasis (height and RWC), photosynthesis (chlorophyll content), and osmotic balance (proline accumulation), but decreased oxidative stress (MDA and H₂O₂). Enhanced activities of critical antioxidant enzymes were observed, particularly in Heydari, which displayed greater enzymatic resilience. At the molecular level, Cs-Pro and Cs-Gly treatments also modulated the expression of stress-responsive genes, such as *TaSOS1* and *TaNHX1*, facilitating improved ion homeostasis, which was reflected in the reduction of Na⁺ accumulation and the maintenance of a favorable Na⁺/K⁺ balance, crucial for cellular stability under saline conditions. While changes in the expression levels of *TaSPDS*, and *TaPxPAO*, confirmed the role of PAs in salt tolerance. The present results underscore the promise of chitosan-based nanoparticles as a sustainable strategy to enhance wheat performance in saline environments. Further research is recommended to explore their applicability across different cultivars and environmental conditions. This innovative strategy represents a significant advancement in sustainable agriculture, offering a pathway to mitigate abiotic stress through nanotechnology.

## Electronic supplementary material

Below is the link to the electronic supplementary material.

Supplementary Material 1

## Data Availability

All the data in the present study are included in this manuscript.

## Abbreviations

Cs-Pro: Chitosan-proline
Cs-Gly: Chitosan-glycine
RWC: Relative water content
MDA: Malondialdehyde
H_2_O_2_: Hydrogen peroxide
PxPAO: Peroxisomal polyamine oxidase
SAMDC: S-adenosylmethionine decarboxylase
SPDS: Spermidine synthase
ADC: Arginine decarboxylase
CREs: *Cis*-acting regulatory elements
NHX: Vacuolar Na^+^/H^+^ antiporter
SOS: Salt overly sensitive
GR: Glutathione reductase
GST: Glutathione S-transferase
GPX: Guaiacol-peroxidase
CAT: Catalase
NT: Nanoparticle treatments
