# Supplementary material for: Enhancing wheat resilience to salt stress through an integrative nanotechnology approach with chitosan proline and chitosan glycine
Source: Sci Rep. 2025 Apr 1;15:11126. doi: 10.1038/s41598-025-91496-w (PMC11961683; doi:10.1038/s41598-025-91496-w)
Supplement: Supplementary file 1 — Supplementary Material 1 [file 41598_2025_91496_MOESM1_ESM.docx]

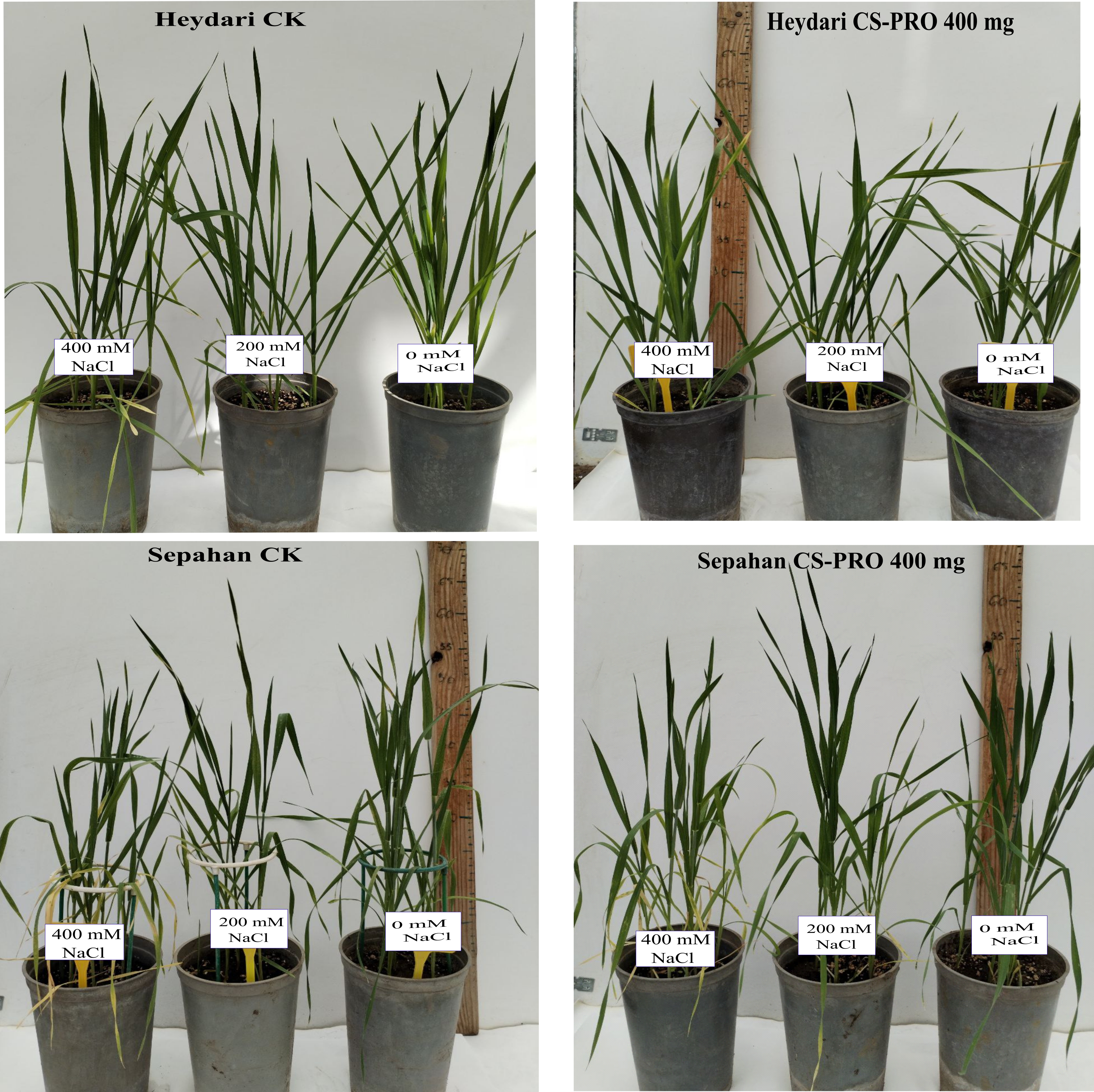


**Fig. S1** The effect of salinity (0, 200 and 400 mM NaCl) after 3 days and protective role of CS-PRO 400 mg on growth performance of two plant cultivars (Heydari and Sepahan).

**Table S1** Analysis of variance the impact of foliar spraying of Cs–Pro and Cs–Gly NPs (0, 200, and 400 mg L^-1^) on morpho-physiological properties of a salt tolerant and a sensitive wheat cultivar (Heydari, Sepahan) under salt stress (0, 200 and 400 mM NaCl).

|  |  | Mean Squares | | | | | | | | | | |
| --- | --- | --- | --- | --- | --- | --- | --- | --- | --- | --- | --- | --- |
| SOV | DF | | RWC | Height | SPAD | MDA | H_2_O_2_ | PRO | CAT | GST | GR | GPX |
| S | 2 | | ** | ** | ** | ** | ** | ** | * | ** | ** | ** |
| R | 3 | | - | - | - | - | - | - | - | - | - | - |
| C | 1 | | ** | ** | ** | ** | ** | ** | ** | ** | ** | ** |
| NT | 4 | | ** | ** | ** | ns | ** | ** | ** | ** | ** | ** |
| S × C | 2 | | ** | ns | ** | ** | ** | ** | ** | * | ** | ns |
| S × NT | 8 | | ** | ** | ** | ** | ** | ** | ** | * | ** | ** |
| C × NT | 4 | | ** | ** | ** | * | ** | ** | ** | ** | ** | ** |
| S × C × NT | 8 | | ** | ** | ** | ** | ** | ** | ** | ** | ** | ** |
| Error | 87 | | - | - | - | - | - | - | - | - | - | - |

| SOV = sources of variations; DF = degree of freedom; RWC= relative water content; MDA= malondialdehyde; PRO = proline; H_2_O_2_ = hydrogen peroxide; CAT = catalase; GST = glutathione-S-transferase; GR = glutathione reductase; GPX = guaiacol-peroxidase; S = salinity; R = replicate; C = cultivar; NT = nanoparticle treatments; ns: not significant; * and **: significant at 5% or 1% probability level, respectively. |
| --- |

**Table S2** Analysis of variance the impact of foliar spraying of Cs-Pro and Cs-Gly NPs (0, 200, and 400 mg L^-1^) on sodium (Na^+^) and potassium (K^+^) content in leaf and root of two wheat cultivars (Heydari, Sepahan) under salt stress (0, 200 and 400 mM NaCl).

|  | **Mean Squares** | | | | | | |
| --- | --- | --- | --- | --- | --- | --- | --- |
| **SOV** | **DF** | Na^+^ leaf | K^+^ leaf | Na^+^/ K^+^ leaf | Na^+^ root | K^+^ root | Na^+^/ K^+^ root |
| S | 2 | ** | ns | ** | ** | ** | ** |
| R | 3 | - | - | - | - | - | - |
| C | 1 | ** | ** | * | ns | ** | ** |
| NT | 4 | ** | ns | ** | ** | ** | ** |
| S × C | 2 | ** | * | ** | ** | ** | ** |
| S × NT | 8 | ** | ** | ** | ** | ** | ** |
| C × NT | 4 | ** | ns | ** | ** | ** | ** |
| S × C × NT | 8 | ** | ** | ** | ** | ** | ** |
| Error | 87 | - | - | - | - | - | - |

ns: not significant; * and **: significant at 5% or 1% probability level, respectively. S = salinity; R = replicate; C = cultivar; NT = nanoparticle treatments.
